# Evaluation of cotton production sustainability and water footprints in the oasis area of southern Xinjiang under climate change

**DOI:** 10.3389/fpls.2026.1789172

**Published:** 2026-03-11

**Authors:** Hongbo Wang, Fuchang Jiang, Xingpeng Wang

**Affiliations:** 1 Modern Agricultural Engineering Key Laboratory at Universities of Education Department of Xinjiang Uygur Autonomous Region, College of Water Hydraulic and Architectural Engineering, Tarim University, Alar, China; 2 Key Laboratory of Comprehensive Utilization of Saline-Alkali Land, Xinjiang Production and Construction Corps, College of Hydraulic and Architectural Engineering, Tarim University, Alar, China; 3 Key Laboratory of Northwest Oasis Water-Saving Agriculture, Ministry of Agriculture and Rural Affairs, Shihezi, China

**Keywords:** AquaCrop model, climate change, cotton yield, TOPSIS, water footprint

## Abstract

**Introduction:**

The sharp increase in temperature and changes in other climatic variables have profoundly impacted cotton growth, posing a significant threat to the stability of cotton yields in the oasis region of southern Xinjiang.

**Methods:**

This study employed the AquaCrop model (Version 6.1) and CMIP6 SSP245BCC-CSM2-MR future climate scenario data to comprehensively evaluate the impacts of different irrigation and mulching methods on cotton water consumption, water footprints (blue and green water footprints), crop yields, and their long-term stability and sustainability from 2021 to 2099.

**Results and discussion:**

Increasing temperatures under future climate change scenarios could significantly reduce cotton water consumption and water footprints, while increase cotton yield, yield stability, sustainability, and overall irrigation water productivity (WP). Compared to the baseline period of 1981–2020, cotton water consumption and water footprints in 2021–2060 and 2061–2099 are expected to decrease by 29.3% and 28.8%, and 41.6% and 42.6%, respectively. Concurrently, cotton yield and WP are projected to experience an increase of 39.8% (50%) and 49.2% (60.25%), respectively. A comprehensive evaluation based on Technique of Order Preference by Similarity to Ideal Solution revealed that under the historical climate conditions from 1981–2020, an irrigation quota of 495 mm for film mulching drip irrigation and 594 mm for filmless drip irrigation exhibited favorable effects on cotton yields but resulted in increased irrigation water consumption. However, under the future climate scenarios for the periods of 2021-2060 and 2061-2099, the elimination of film mulching and a reduction in irrigation quotas are not expected to have a detrimental impact on the sustainability and stability of cotton yields. This study provides valuable insights for enhancing the resilience and productivity of cotton in response to climate change in southern Xinjiang and analogous regions, which hold significant for policymakers to formulate strategies for the sustainable development of agriculture and plan the allocation of water resources in the future.

## 1 Introduction

Climate change has garnered increasing global attention, with human activities contributing a notable 1.25 °C rise in global temperatures. Alarmingly, current emission trajectory indicates that the temperature change can exceed 1.5 °C within a mere decade (Matthews and Wynes, 2022). By 2100, it is predicted that a substantial 4.8 °C increase in atmospheric temperatures, accompanied by reduced precipitation in arid regions and heightened precipitation in humid areas (IPCC, 2023). Crop yields are intricately tied to climatic conditions (Ahmadi et al., 2021), and the climate determines the production and productivity of agricultural activities (Babaee et al., 2021). Temperature, CO_2_, and the occurrence of different extreme events (drought, heat) might significantly affect crop growth and development, yield, and the strategies employed in field management (Cammarano and Tian, 2018). A previous report suggested that the annual growth rate of global agricultural production is expected to decrease, with a projected decline of 1.5% per year by 2030 and an additional drop of 0.9% by 2050, which contrasts with the historical trend of a 2.3% annual growth rate observed since 1961 (Dubey and Sharma, 2018). Therefore, deepening our understanding of the impact of future climate changes on agricultural systems and implementing proactive strategies to enhance water use efficiency and productivity in agriculture are imperative responses to challenges like climate change, growing population pressures, and limited water resources (Liu et al., 2023a).

Research indicates that drought induced by climate change serves as a primary driver of crop yield reduction (FAO, 2017). During the period from 2001 to 2024, adverse weather conditions led to a global loss of approximately 804 million tonnes of rice, maize, wheat, and soybean in major grain-producing regions (Noia et al., 2025). Maize accounted for the largest share of this loss (estimated at 336 million tonnes), followed by wheat (244 million tonnes). By 2050, the combined drought-related loss for maize, soybean, rice, and wheat is projected to be below 2%, while soybean alone may experience a loss of up to 3.6%. Although the global average change appears modest, 62 countries suffered maximum yield losses exceeding 10%, with 24 countries experiencing losses over 20% (Kraklow et al., 2026). Concurrently, climate change is reshaping agricultural land suitability, exerting profound impacts on water resources, carbon emissions, and crop productivity. Under the SSP1-2.6 and SSP2-4.5 scenarios, highly suitable agricultural areas are projected to increase by 27.7 million hectares and 18.7 million hectares, respectively, during 2021–2060, followed by a slight decline in 2061–2100. In contrast, under the SSP5-8.5 scenario, these highly suitable areas are expected to continue shrinking, dwindling to only 24 million hectares by 2100 (Guo et al., 2026). Because climate change manifests in different ways across different regions, its impact might fluctuate due to localized climate changes. In addition, different crops may not respond uniformly to climate change, as their responses can diverge owing to changes in their sensitivities to changes in temperature, precipitation, and CO_2_. Therefore, the impacts of climate change can differ based on specific crop, geographic location, and field management practices, making it impractical to generalize across different regions and crops, thus necessitating tailored, site-specific impact assessments (Rashid et al., 2019).

Considering the intricate nature of farmland ecosystems and the limitations of conducting field experiments, crop models combined with long-term weather data provide an opportunity for assessing yield variability by simulating diverse array of potential scenarios (Masasi et al., 2020). Utilizing a coupled and downscaled meteorological data from 31 global climate models for two representative concentration pathways in China, Jiang et al. employed the AquaCrop model to quantify changes in corn yield and assess alterations in water footprint (WF) (Jiang et al., 2022). Through simulating the impact of climate change on crop production in food insecure areas, Alvar-Beltrán et al. (2021) reported the potential jeopardy faced by the productivity of major crops, which could have significantly impact on both food security and farmers’ incomes (Jorge et al., 2023). Based on eight climate models, Voloudakis et al. applied the AquaCrop model in conjunction with discriminant function analysis to predict the impact of climate change on cotton yield in Greece (Voloudakis et al., 2015). By applying the AquaCrop model, Bird et al. (2016) demonstrated that, under the future climate conditions spanning 2040 to 2070, crop yields would experience a substantial decrease in comparison to a scenario where no adaptation measures were implemented.

Xinjiang’s distinctive climatic environment provides favorable conditions for cotton cultivation, making it China’s largest production base for high-quality cotton (Wang et al., 2020). Statistical data show that Xinjiang accounted for 83.22% of the nation’s total cotton planting area and 90.20% of its total output. Specifically, the region’s cotton planting area reached 2.50 million hectares, with an output of 5.39 million tons (National Bureau of Statistics, 2022). However, for southern Xinjiang, characterized by limited water resources and low agricultural water efficiency, the prospect of rising temperatures and diminishing precipitation in the future might bring new challenges to the sustainable production of crops in the region (Li et al., 2019). Faced with increasingly serious water shortages, climate change and its uncertainty, there is an imperative requirement to improve agricultural water efficiency and productivity as a means to mitigate detrimental environmental consequences (Alvar-Beltrán et al., 2021). In the past few decades, researchers have increasingly focused on the impact of climate change on crop production (Li et al., 2020a) and have found that crop growth and yield responses to climate change can be modulated through the adoption of various field management practices, including different tillage management strategies (Li et al., 2020b), adjustments in planting date, and the selection of efficient water-saving irrigation technologies and methods.

In recent years, the average temperature in Xinjiang has increased at a rate of 0.3 °C per decade (Li et al., 2020a). The substantial rise in temperature and changes in other climate variables have affected cotton growth and threatened the stability of cotton yield in Xinjiang (Li et al., 2019). Simultaneously, the complexity and uncertainty surrounding the intensity and direction of the impact of climate change on crop yield can lead to either net positive or negative outcomes (Zhao et al., 2018). Therefore, it is imperative to enhance the understanding of future climate’s impacts on agricultural systems to facilitate improved crop management and the mitigation of forthcoming climate change effects (Jin et al., 2017). This study evaluated the impact of different irrigation and mulching methods on cotton water consumption, yield, and water footprints (blue and green water footprints), analyzes the sustainability and stability of cotton production under the temperature increase, and determines the cultivation patterns and irrigation water strategies under different climate conditions. The research results provide a reference for improving the tolerance and productivity of cotton in response to climate change in southern Xinjiang and similar areas, which is of great significance to policymakers in formulating future sustainable agricultural development strategies and water resource planning.

## 2 Materials and methods

### 2.1 Experimental site

The experimental site, situated in Alar City within the First Division of the Xinjiang Production and Construction Corps (81°17′56″E, 40°32′36″N, 1100 m above sea level), exhibits typical arid continental climate traits, characterized by a dry climate and abundant thermal resources. The average annual precipitation is only 50 mm, and the annual evaporation is 2218 mm. The average frost-free period is 207 days, and the average annual temperature is 11.3 °C. This area is representative of cotton production, encompassing approximately 160,000 hectares dedicated to cotton cultivation, accounting for 6.41% of Xinjiang’s total cotton plantation area.

### 2.2 Description of the AquaCrop model

The AquaCrop model elucidates the interaction between crops and water in the soil–crop–atmosphere system, and detail information about the model is available in Steduto et al (Steduto et al., 2009). Briefly, the model encompasses four sub-models: (1) an atmospheric sub-model consisting of rainfall, reference evapotranspiration, and carbon dioxide concentration; (2) a crop sub-model involving crop growth, development, and yield; (3) a management sub-model including irrigation and field management practices; and (4) a soil sub-model dealing with soil water balance (Irmak et al., 2022).

The AquaCrop model simulates crop yield following these steps: (1) crop development; (2) crop transpiration; (3) biomass production; and (4) yield formation (Alvar-Beltrán et al., 2021). The AquaCrop model calculates actual crop evapotranspiration (ETc act) and expresses it as transpiration (T) and evaporation (E) from the soil surface, based on the daily soil water balance. The standardized crop water productivity (WP*) model was used to estimate biomass weight and the ratio of transpiration to evapotranspiration (T/ETc act) during the crop growth period (Li et al., 2019). The final yield was determined by the product of the biomass and the harvest index (Li et al., 2019). The model is relatively simple but very robust in simulating different plant growth and development processes and has been widely used in climate change research to simulate the effects of rainfall, temperature, and carbon dioxide (Muluneh et al., 2015).

### 2.3 Data collection

#### 2.3.1 Atmosphere sub-model

##### 2.3.1.1 Historical climate

Meteorological data collected from 2017–2020 for a field experiment were sourced from agricultural meteorological stations positioned at the experimental site, encompassing temperature, humidity, wind speed, rainfall, and solar radiation measurements. Historical meteorological data from 1981–2016 were gathered from the China Meteorological Administration. The daily reference crop evaporation (ETo) was calculated using the Penman–Monteith model recommended by the Food and Agriculture Organization of the United Nations (FAO) (Allen et al., 1998). The CO_2_ concentration data adopted the model default value (RCP4.5).

##### 2.3.1.2 Future climate

Monthly meteorological data of the BCC-CSM2-MR climate model from 2021 to 2100 were downloaded from the CMIP6 Global Climate Research Program Center (https://esgf-node.llnl.gov/search/cmip6/), including precipitation, radiation, daily maximum temperature, daily minimum temperature, and relative humidity. Using the WGEN random weather generator, the NWAI-WG statistical downscaling method was used to perform temporal downscaling on monthly grid data from the SSP245BCC-CSM2-MR climate model under the CMIP6 climate scenario, and daily meteorological data from 2021 to 2100 were generated (Köster et al., 2026).

#### 2.3.2 Crop sub-model

**Figure 1:**
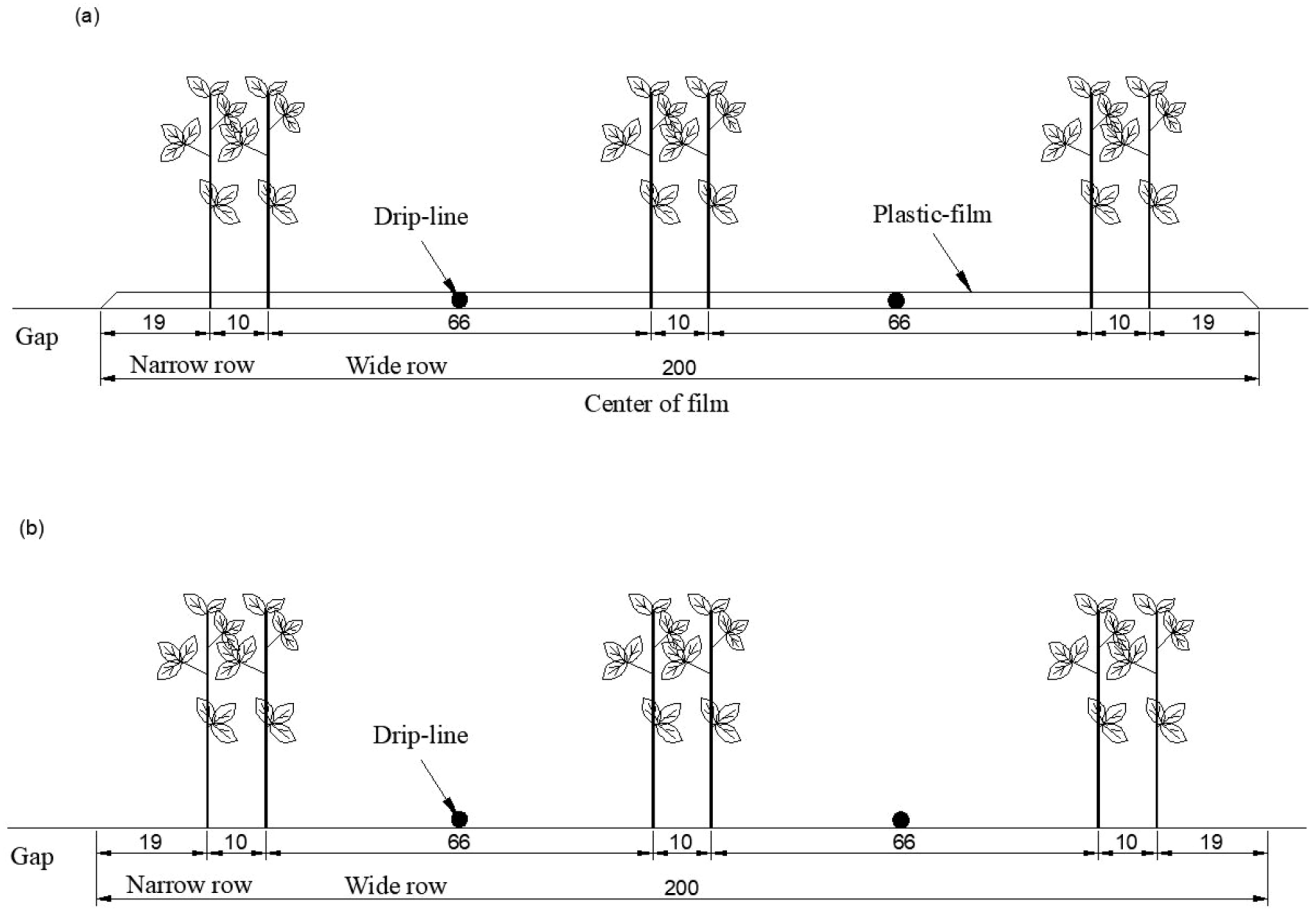
Schematic diagram of cotton planting patterns under **(a)** plastic mulch and **(b)** without mulching.

To alleviate the adverse impacts of mulch residue on the soil environment and promote sustainable cotton production, filmless drip irrigation has become a novel and sustainable farming model for the future of agriculture (Wang et al., 2023). Fan et al. (2019) conducted comprehensive field experiments on film mulching drip irrigation conditions in 2017–2018. Wang et al. (2023) calibrated and validated the crop parameters of the AquaCrop model for film mulching drip irrigation based on two-year field experiment data (2017–2018), thus confirming the applicability and reliability of the model. These crop parameters are shown in **Table 1**. Through comprehensive field experiments under filmless drip irrigation conditions from 2018 to 2019, Wang et al. (2021) used field experiment data to calibrate and validate the crop parameters specific to filmless drip-irrigated cotton (**Table 1**). At the same time, the filmless drip irrigation system for cotton in southern Xinjiang was optimized based on the AquaCrop model. The cotton plantation pattern during 2017–2019 was 1 film, 3 columns and 6 rows, and row spacing was 10 cm + 66 cm + 10 cm + 66 cm + 10 cm. The distance between two plants in the same row was 10 cm, and the planting density was 240,000 plants ha^−1^. The cotton plantation patterns under the two mulching conditions are shown in **Figures 1a, b**, respectively.

**Table 1:** Crop parameters of the AquaCrop model for cotton with plastic mulch and drip irrigation (FMDI) and drip irrigation without mulching (WMDI).

Description and unit	Value	
	FMDI	WMDI
Canopy cover per seeding at 90% emergence, %	1.2	1.0
Canopy growth coefficient, %/d	6.8	10.3
Decline in crop coefficient after reaching, %	90	90
Canopy decline coefficient at senescence, %/d	5.2	8
Maximum crop coefficient	1.15	1.1
Maximum effective rooting depth	0.8	0.65
Water productivity normalized for ETo and CO_2_, g/m^2^	18	19
Reference harvest index, %	34	41
Leaf growth threshold p-upper	0.35	0.25
Leaf growth threshold p-lower	0.65	0.55
Stomatal conductance threshold p-upper	0.35	0.55
Soil water depletion threshold for senescence acceleration	0.60	0.88

#### 2.3.3 Management sub-model

**Figure 2:**
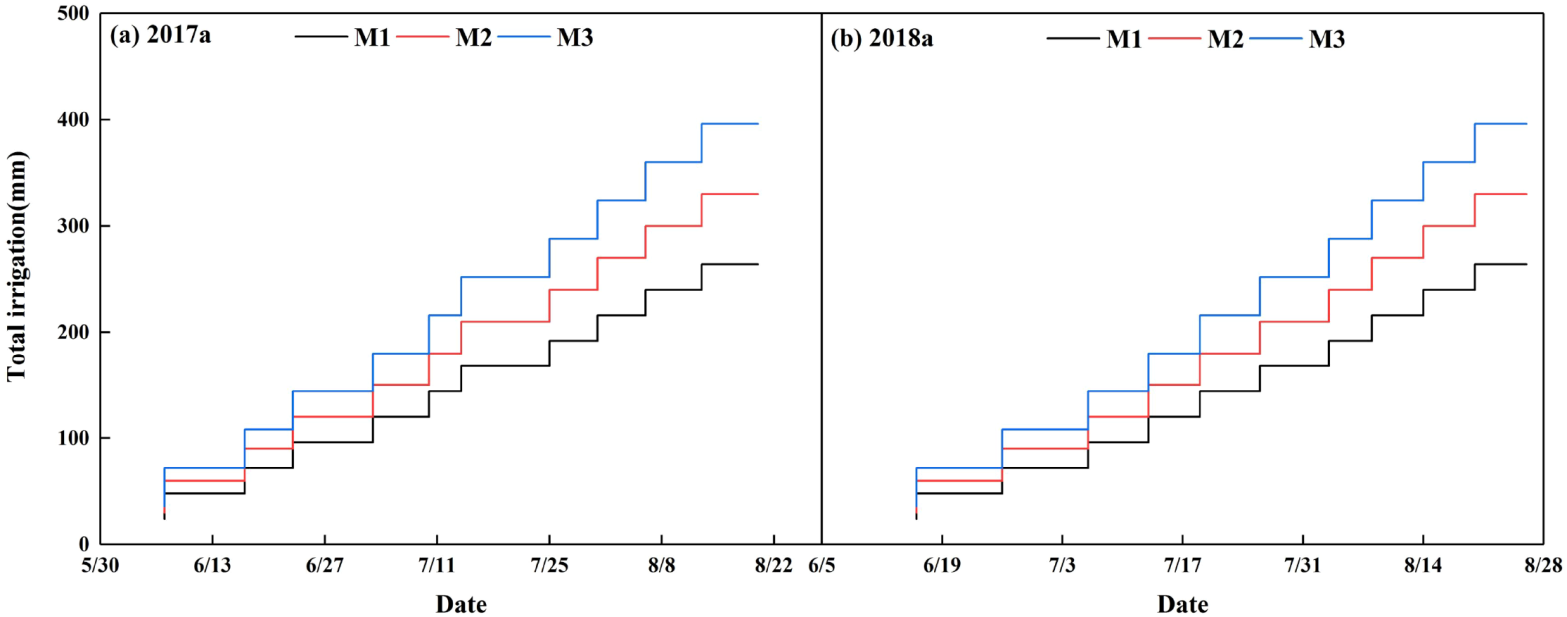
Cotton irrigation system under film mulching drip irrigation conditions in 2017–2018.

**Figure 3:**
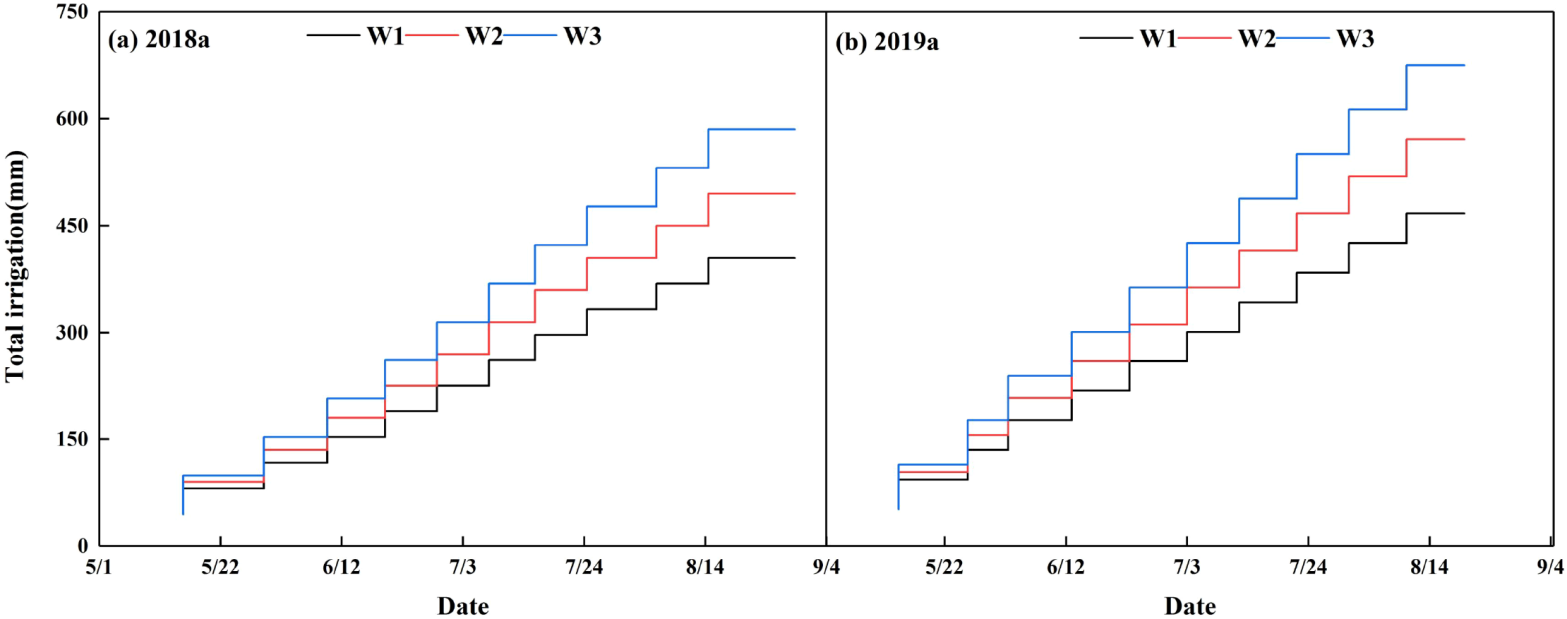
Cotton irrigation system under filmless drip irrigation conditions in 2018–2019.

The management sub-model primarily included sub-models for irrigation and field management practices. Drip irrigation was used for cotton irrigation from 2017 to 2019. **Figures 2a, b** show the irrigation schedules for three water treatments of 24 mm (M1), 30 mm (M2), and 36 mm (M3) under film mulching conditions in 2017 and 2018, respectively (Fan et al., 2019). **Figures 3a, b** illustrate the irrigation regimes for three water treatments of 36 mm (W1), 45 mm (W2), and 54 mm (W3) under filmless conditions in 2018 and 2019, respectively (Wang et al., 2021, 2023). Field management data, including mulching, pest management, fertilization, and weed control, were gathered based on local cotton field management practices, contributing to the establishment of a comprehensive field management database.

#### 2.3.4 Soil sub-model

Before cotton sowing in 2017–2019, soil samples were collected using an auger at the depths of 0–20, 20–40, 40–60 and 60–80 cm and soil physical properties were measured in the Key Laboratory of Crop Water Use and Regulation of the Ministry of Agriculture and Rural Affairs. The soil is classified as sandy loam, consisting of clay particles at 2.62%, silt particles at 41.78%, and sand particles at 55.60%. The soil bulk density, field water capacity, saturated water content, and wilting water content were 1.6 g cm^−3^, 0.2 g g^−1^, 0.3 g g^−1^, and 0.1 g g^−1^, respectively. The soil water effects of capillary rise were not simulated because the groundwater table (below 3.5 m) was below the rooting zone (Steduto et al., 2012).

### 2.4 Scenario simulation

In northwestern China, by the end of the 21^st^ century (2081–2100), under extreme conditions, it is anticipated that the average temperature may undergo a change of 1.5–2.0 °C, with a corresponding alteration in average precipitation ranging between 10–20% (IPCC, 2023). This study conducted an analysis of two cultivation modes and six irrigation levels based on the predicted future temperature changes. At present, the water quota for cotton cultivation with film mulching drip irrigation in southern Xinjiang was about 30 mm, and the water quota for cotton cultivation using filmless drip irrigation was about 45 mm. While there were slight differences observed in each region, the differences were relatively insignificant. In pursuit of this objective, we established a range of irrigation treatments, denoted as I1 to I6, utilizing the 30 mm irrigation quota. Additionally, two mulching methods were employed: traditional plastic film mulching (F) and without mulching (N) (**Table 2**).

**Table 2:** Simulation scenarios.

Year	Treatment	Mulching pattern	Irrigation rate (mm)/irrigation quota (mm)	Treatment	Mulching pattern	Irrigation rate (mm)/irrigation quota (mm)
1981–2020	F_1_	Film mulching (F)	18/198	N_1_	Without mulching (N)	18/198
	F_2_		24/264	N_2_		24/264
	F_3_		30/330	N_3_		30/330
	F_4_		36/396	N_4_		36/396
	F_5_		45/495	N_5_		45/495
	F_6_		54/594	N_6_		54/594
2021–2060	F_7_		18/198	N_7_		18/198
	F_8_		24/264	N_8_		24/264
	F_9_		30/330	N_9_		30/330
	F_10_		36/396	N_10_		36/396
	F_11_		45/495	N_11_		45/495
	F_12_		54/594	N_12_		54/594
2061–2099	F_13_		18/198	N_13_		18/198
	F_14_		24/264	N_14_		24/264
	F_15_		30/330	N_15_		30/330
	F_16_		36/396	N_16_		36/396
	F_17_		45/495	N_17_		45/495
	F_18_		54/594	N_18_		54/594

### 2.5 Water footprint

The components of the crop production water footprint (WF) included blue water (WF_B_) and green water (WF_G_) (Ahmadi et al., 2021), which were used to reflect the type and amount of crop production water and were calculated using Equations 1–4.

(1) $$WF_{B}=\left(ET-P\right)/Y$$

(2) $$WF_{G}=\left(P\times 10\right)/Y$$

(3) $$T_{WF}=WF_{G}+WF_{B}$$

(4)  ET=ΔS+P+I

where P is actual precipitation consumed by crops (mm), ET is the evapotranspiration of each plant during the growth period (mm), ΔS is change in soil moisture content from the start to the end of the period (mm); I is irrigation water supply received by the cotton field (mm)and Y is cotton yield (t ha^−1^), respectively.

### 2.6 Irrigation water productivity

Irrigation water productivity (WP, kg m^-3^) was determined according to the method described by You et al. (2022) and Wang et al. (2024). They was calculated using Equation 5.

(5) WP=Y/I

where Y is seed cotton yield (kg ha^−1^) and I is the volume of irrigation water in the cotton field during the growth period (m^3^ ha^−1^).

### 2.7 Evaluation of cotton yield sustainability and stability

This study used the sustainability index (SYI) to evaluate the sustainability of cotton yield and the coefficient of variation (CV) to evaluate the stability of cotton yield. They were calculated using Equations 6, 7, respectively.

(6) $$SYI=\left(Y_{mean}-S\right)/Y_{max}$$

(7) $$CV=S/Y_{mean}\times 100$$

where Y_mean_ is the average yield (t ha^−1^), S is the standard deviation of the yield, and Y_max_ is the maximum yield (t ha^−1^), respectively.

### 2.8 Comprehensive evaluation

The optimal strategy of irrigation and mulching for cotton yield under climate change was evaluated by using TOPSIS. The calculation was performed following Hamani et al. (2023) and Wang et al. (2024).

### 2.9 Statistical evaluation criteria

This study used statistical error standards, including the coefficient of determination (R^2^), root mean square error (RMSE), normalized root mean square error (NRME), and synergy index (d), for evaluation. The detailed calculation process was reported by Wang et al. (2023).

## 3 Results

### 3.1 Future temperature changes

**Figure 4:**
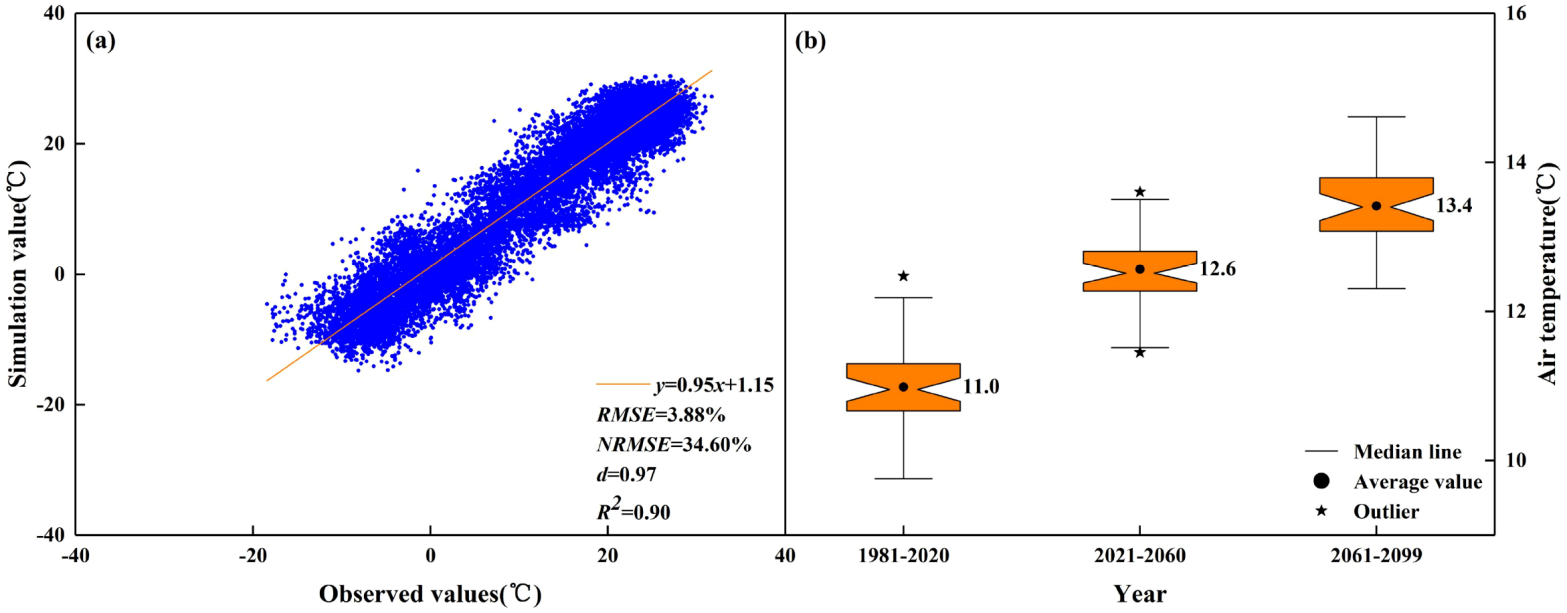
Historical temperature fitting and future temperature prediction. **(a)** Observed and simulated values of historical meteorological data; **(b)** Air temperature changes under different climate scenarios.

In this study, the historical meteorological data from 1981 to 2020 was used to validate the predictions of average temperature changes produced by the CMIP6 series BCC-CSM2-MR model. **Figure 4a** shows a strong correlation between the measured and simulated values of the average temperature, with *R^2^* of 0.9. The *RMSE* was 3.9%, the *NRMSE* was below 34.6% and *d* was 1.0, indicating the high reliability of NWAI-WG for predicting future meteorological changes. The prediction results showed that, in comparison to the period from 1981 to 2020, the average atmospheric temperatures in 2021–2060 and 2061–2099 could increase by 1.6 °C and 2.4 °C, respectively (**Figure 4b**).

### 3.2 Cotton biomass, yield, and yield stability and sustainability

**Figure 5:**
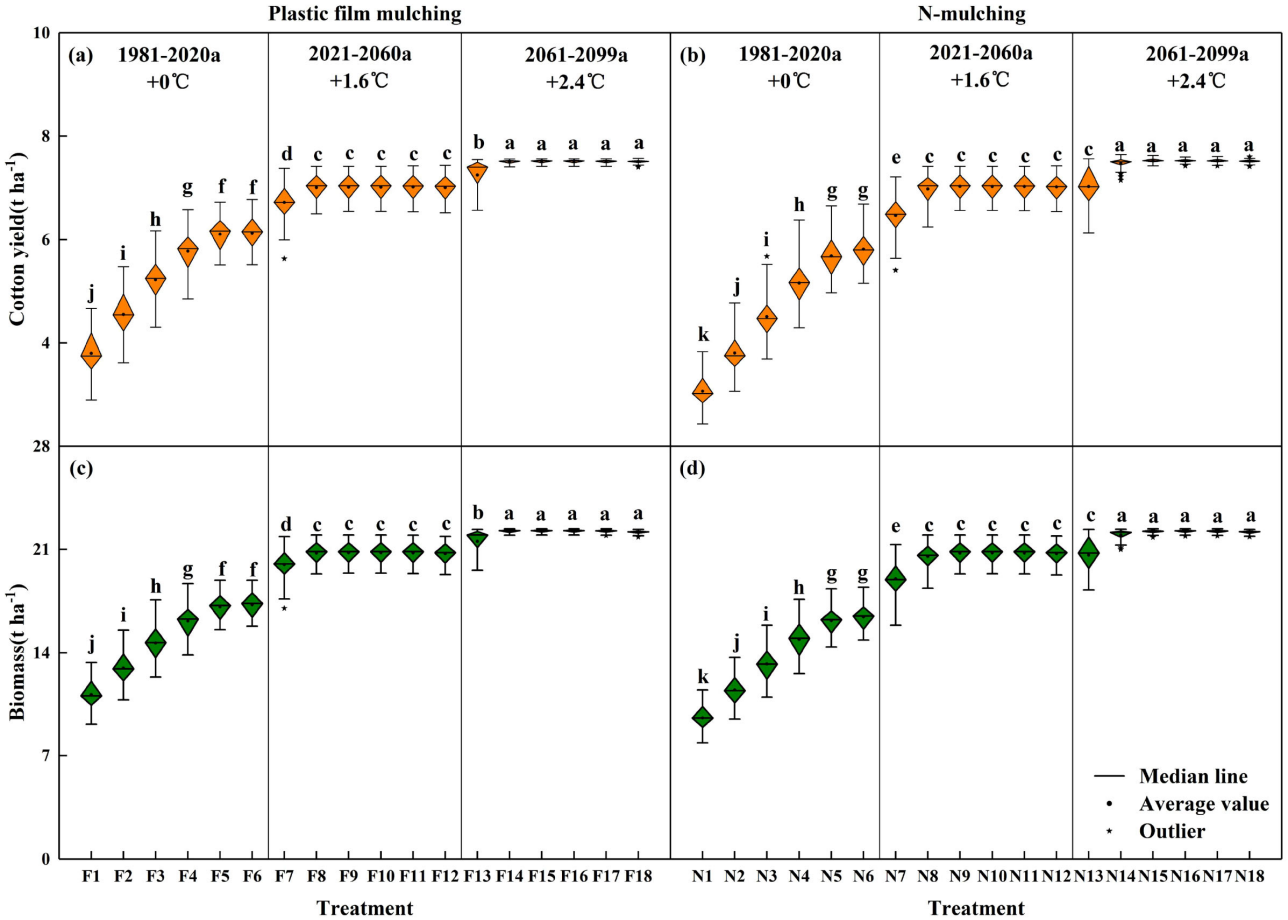
Cotton biomass and yield under different scenarios. **(a, c)** represent the yield and biomass of cotton under mulch drip irrigation; **(b, d)** represent the yield and biomass of cotton under non-mulch drip irrigation. Lowercase indicates the difference significance among treatments at 0.05 level.

**Figure 5** presents cotton biomass and yield under different scenarios. Under historical climate conditions in 1981–2020 (+0 °C), the cotton yield displayed a positive correlation with increasing irrigation quotas, with the high irrigation quota treatment significantly outperforming other treatments (P<0.05). The yield in F5–F6 increased by 11.2 and 46.5% compared to F3–F4 and F1–F2 (film mulching), respectively. The yield in N5–N6 increased by 19.0 and 67.4%, respectively, compared to N3–N4 and N1–N2 (without mulching). Under similar irrigation quotas, the cotton yield with film mulching treatment increased significantly by 12.6% compared to the treatment without mulching, with the difference between treatments being statistically significant. Under the climate conditions of 2021–2060 (+1.6 °C) and 2061–2099 (+2.4 °C), the cotton yield in the F7 and N7 treatments was significantly lower than those in the other treatments, while the mulching method had little effect on cotton yield (P<0.05). In general, there was a noteworthy increase in cotton yield with rising temperatures. Compared with 1981–2020 (+0 °C), the cotton yield in 2021–2060 (+1.6 °C) and 2061–2099 (+2.4 °C) increased by 32.4 and 41.9% with film mulching and by 48.2 and 59.1% without mulching. Crop aboveground biomass is an important indicator for evaluating adaptability to environmental conditions. In this study, the simulated values of cotton aboveground biomass and yield presented similar patterns of change (**Figures 5c, d**).

As shown in **Table 3**, the stability and sustainability of cotton yield under the two mulching methods showed an upward trend with an increase in irrigation quota and temperature. The coefficients of variation ranged from 0.5 to 11.8 for cotton cultivation with film mulching and from 0.5 to 12.0 for cotton cultivation without mulching. Meanwhile, the sustainability indices ranged from 0.7 to 1.0 for cotton cultivation with film mulching and from 0.7 to 1.0 for cotton cultivation without mulching.

**Table 3:** Comparison of variation coefficient and sustainability index of annual cotton yield in different scenarios.

Treatment	Coefficient of variation	Sustainability index	Treatment	Coefficient of variation	Sustainability index
F1	11.75	0.72	N1	12.02	0.70
F2	10.50	0.74	N2	11.81	0.70
F3	9.54	0.77	N3	11.27	0.70
F4	7.79	0.81	N4	9.92	0.73
F5	5.66	0.86	N5	7.34	0.79
F6	5.52	0.85	N6	6.59	0.81
F7	5.90	0.86	N7	6.21	0.84
F8	3.86	0.91	N8	4.13	0.90
F9	3.74	0.91	N9	3.69	0.91
F10	3.74	0.91	N10	3.70	0.91
F11	3.73	0.91	N11	3.70	0.91
F12	3.74	0.91	N12	3.71	0.91
F13	4.24	0.92	N13	5.55	0.88
F14	0.54	0.99	N14	1.42	0.96
F15	0.48	0.99	N15	0.60	0.98
F16	0.47	0.99	N16	0.53	0.99
F17	0.47	0.99	N17	0.52	0.98
F18	0.48	0.99	N18	0.51	0.98

### 3.3 Cotton water consumption, water footprint, and irrigation water productivity

**Figure 6:**
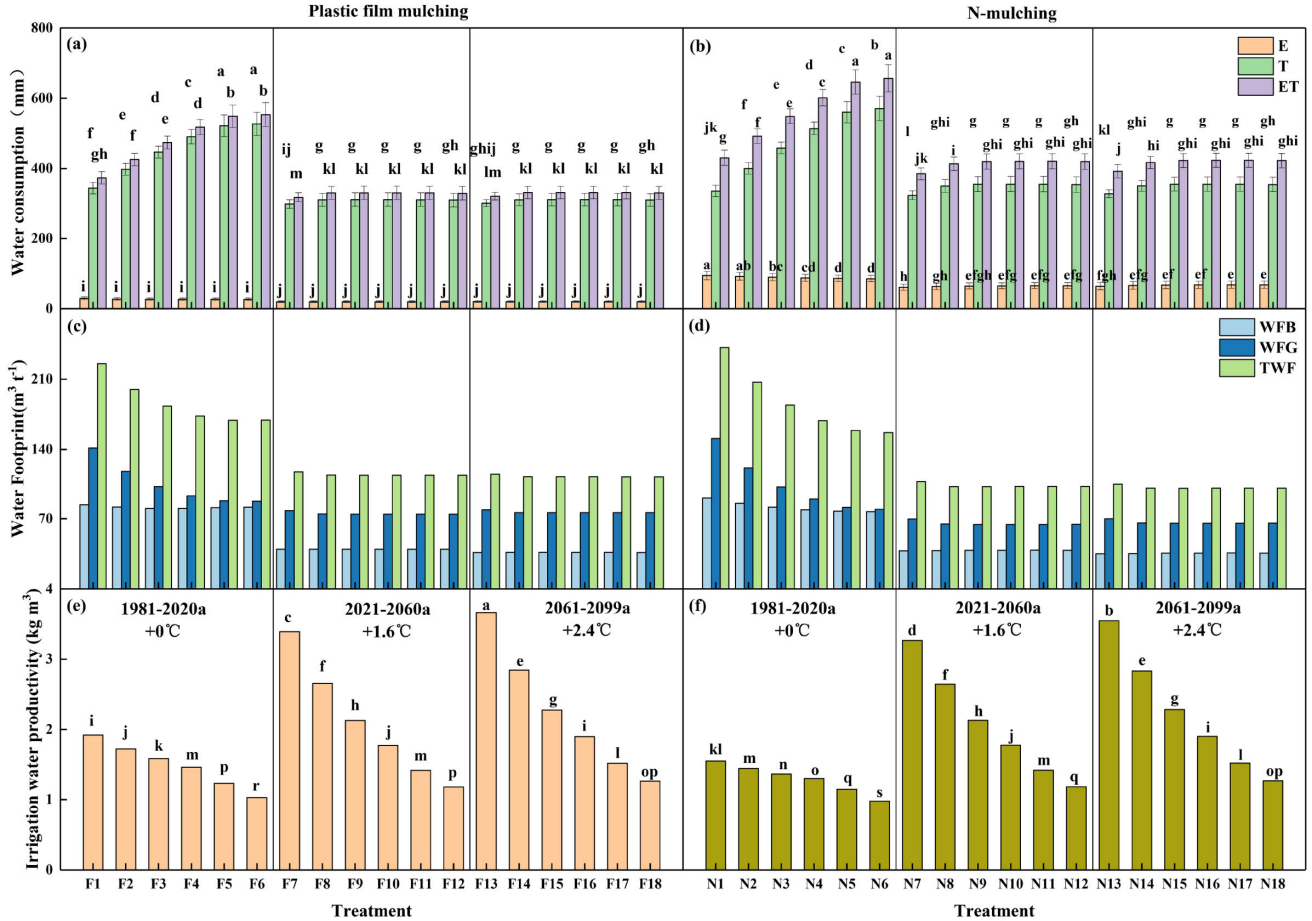
Water consumption and irrigation water productivity of cotton under different treatments. **(a, c, e)** represent the water consumption, water footprint, and irrigation water productivity of cotton under mulch drip irrigation, respectively; **(b, d, f)** represent the water consumption, water footprint, and irrigation water productivity of cotton under non-mulch drip irrigation, respectively. Lowercase indicates the difference significance among treatments at 0.05 level. The short line represents the standard error.

**Figures 6a, b** show cotton water consumption under different scenarios. For the historical climate conditions in 1981–2020 (+0 °C), the water consumption of cotton with both mulching methods showed an upward trend with the increase in irrigation quota. The water consumption of cotton in F5 and F6 was 548.3 and 553.3 mm, respectively, which were significantly higher than those in the F1–F4 treatments. The water consumption of cotton in the N5 and N6 treatments was 565.3 and 574.1 mm, respectively, which were significantly higher than those in the N1–N4 treatments. Additionally, the water consumption of cotton in the N4–N6 treatments was significantly higher than that in the F4–F6 treatments (P<0.05), with an average increase of 11.8%. Under the climate conditions of 2021–2060 (+1.6 °C) and 2061–2099 (+2.4 °C), the water consumption of cotton significantly decreased, by 32.0 and 31.7% with film mulching, and by 26.6 and 26.0% without mulching. For the similar periods (i.e., 2021–2060 and 2061–2099), the water consumption of cotton without mulching treatments exhibited a significant increase, surpassing that of the film mulching treatments by 10.2% and 10.7%, respectively.

The water footprints of cotton production under different scenarios are shown in **Figures 6c, d**. The total water footprint, blue water volume, and green water volume of cotton production with mulching treatments were higher than those without mulching treatments, increasing by 15.1–16.6% in 1981–2020, 4.8–12.7% in 2021–2060 and 4.7–13.4% in 2061–2099, respectively. The overall water footprint of cotton production exhibited a decreasing trend in respond to climate change. The total water footprint of cotton production was 186.6, 114.8, and 113.0 m^3^ t^−1^ in 1981–2020, 2061–2099, and 2061–2099, respectively, of which the blue water volume accounted for 44.1%, 34.4%, and 32.2%, respectively. The total water footprint in the treatments without mulching was 216.0, 120.4 and 118.3 m^3^ t^−1^ in 1981–2020, 2061–2099, and 2061–2099, respectively, of which the blue water volume accounted for 44.7%, 37.1%, and 34.5%, respectively.

For the historical climate conditions from 1981–2020 (see **Figures 6e, f**), WP of cotton with the two mulching methods showed a strong downward trend with the increase in the irrigation quota. In the F1–F6 treatments, WP ranged from 1.9 to 1.0 kg m^−3^, while in the N1–N6 treatments, it ranged from 1.6 to 1.0 kg m^−3^. Simultaneously, the WP of cotton with film mulching treatments was significantly higher compared to that without mulching treatments, with an increase of 14.9%. For the future climate conditions in 2021–2060 and 2061–2099, the WP of cotton exhibited a significant downward trend with the increase in irrigation quota. Under the two mulching methods, the WP of cotton in the F13 and F25 treatments (film mulching) and the N13 and N25 treatments (without mulching) were significantly higher the other treatments.

### 3.4 Comprehensive evaluation of TOPSIS

The comprehensive evaluation using the TOPSIS method, which takes into account biomass, yield, soil evaporation, water consumption, WP, and water footprint is shown in **Table 4**. Biomass, yield, water consumption, and WP were considered as positive indicators, while soil evaporation and the total water footprint were used as reverse indicators for weight calculation using the entropy weight method. The weights of biomass, yield, soil evaporation, water consumption, WP, and the water footprint were 8.1%, 6.9%, 15.6%, 39.2%, 24.5%, and 5.7%, respectively. Among the indicators, soil water consumption held the highest weight at 39.2%, while the total water footprint had the lowest weight of 5.7%. For the historical climate conditions in 1981–2020 (+0 °C), the top-performing mulching treatments, as determined by TOPSIS scores and rankings, were F5 with film mulching and N6 without mulching treatments, both of which were ranked 1^st^. For the future climate conditions in 2021–2060 (+1.6 °C), the mulching treatments that performed the best were F7 with film mulching and N7 without mulching. For the future climate conditions in 2061–2099 (+2.4 °C), the top-performing treatments were F13 with film mulching and N13 without mulching.

**Table 4:** Comprehensive evaluation based on TOPSIS.

Treatment	Euclidean distances		Comprehensive score index	TOPSIS rank	Treatment	Euclidean distances		Comprehensive score index	TOPSIS rank
	d+	d-				d+	d-		
F1	0.70	0.41	0.37	34	N1	0.87	0.18	0.17	36
F2	0.59	0.48	0.45	22	N2	0.76	0.30	0.28	35
F3	0.52	0.57	0.52	7	N3	0.68	0.43	0.39	33
F4	0.47	0.66	0.58	3	N4	0.63	0.55	0.47	19
F5	0.48	0.73	0.60	1	N5	0.61	0.66	0.52	9
F6	0.52	0.73	0.59	2	N6	0.63	0.68	0.52	8
F7	0.63	0.71	0.53	5	N7	0.63	0.59	0.48	14
F8	0.63	0.65	0.51	11	N8	0.60	0.55	0.48	17
F9	0.66	0.61	0.48	15	N9	0.63	0.51	0.45	23
F10	0.69	0.59	0.46	20	N10	0.66	0.48	0.42	28
F11	0.73	0.58	0.44	26	N11	0.69	0.47	0.40	31
F12	0.75	0.57	0.43	27	N12	0.72	0.46	0.39	32
F13	0.62	0.76	0.55	4	N13	0.61	0.65	0.52	10
F14	0.61	0.69	0.53	6	N14	0.59	0.59	0.50	12
F15	0.65	0.65	0.50	13	N15	0.61	0.55	0.47	18
F16	0.68	0.62	0.48	16	N16	0.64	0.52	0.45	25
F17	0.71	0.61	0.46	21	N17	0.68	0.50	0.42	29
F18	0.74	0.60	0.45	24	N18	0.71	0.49	0.41	30
w_B_=8.13%, w_Y_=6.94%, w_E_=15.57%, w_ET_=39.19%, w_WP_=24.50%, w_WF_=5.67%									

w_B_, w_Y_, w_E_, w_ET_, w_WP_ and w_WF_ are the weights of biomass, yield, soil evaporation, water consumption, irrigation water productivity and water footprint, respectively.

## 4 Discussion

Recently, the World Climate Research Programme (WCRP) Working Group on Coupled Modelling (WGCM) launched the Coupled Model Intercomparison Project Phase 6 (CMIP6). CMIP6 has the largest number of participating models, the most abundant designed numerical experiments, and the largest amount of simulation data since the implementation of CMIP over the past two decades. Its climate system simulations have shown improved agreement with observed values, resulting in reduced uncertainties when comparing different models (Kim et al., 2020). Crop model simulations, combined with future climate scenario data from GCMs, are widely used to assess crop responses and adaptations to future climate change (Xiao et al., 2020). Compared with 1981–2020, we found that the average atmospheric temperatures in 2021–2060 and 2061–2099 increased by 1.6 °C and 2.4 °C (SSP2-45), respectively. Our findings are similar to Tan et al., who reported that the average temperature of northwestern China in 2081–2100 could vary between 1.5 °C and 2.0 °C under extreme conditions (Tan et al., 2018). Moreover, The IPCC (2023) states that the emission of greenhouse gases has resulted in global warming, elevating the global surface temperature by 1.1°C in 2011-2020 relative to the 1850–1900 baseline. Persistent emissions will induce additional warming. Based on the evaluated scenarios and modeled pathways, the best estimate projects that the global temperature increase will attain 1.5°C within the near-term period of 2021-2040.

The growth rate and yield of cotton are primarily determined by temperature (Li et al., 2020a), while also being influenced by the application levels of irrigation, pesticides, and fertilizers (Zhang et al., 2023). The cotton biomass and yield in both mulching models from 1981 to 2020 demonstrated an upward trend with an increase in the irrigation quota. It is indicated that irrigation can increase the photosynthetic products of cotton and improve the rate of dry matter accumulation of cotton (Hou et al., 2021). However, the irrigation quota of 495–594 mm did not significantly increase the cotton yield, indicating that both higher and lower irrigation amounts are not conducive to achieving high cotton yields (Wang et al., 2016; Hu et al., 2018; Xie et al., 2020). Excessive irrigation causes fertilizer leaching, which in turn reduces fertilizer absorption and utilization efficiency, thereby limiting the reproductive growth of cotton plants. Appropriate irrigation practices are more favorable for increasing cotton yield (Wang et al., 2024). Furthermore, the increase in biomass accumulation cannot be converted into final yield. Excessive accumulation of biomass in the cotton root and stem systems reduces cotton yield, limits boll formation, and results in the wastage of water resources and fertilizers (Wang et al., 2022). The cotton yield in the film mulching treatment was significantly higher than that without the mulching treatment, showing an average increase of 12.6% compared to the non-mulching treatment. This is because mulching improves water use efficiency and crop yield by reducing soil water evaporation, regulating soil temperature, and promoting photosynthesis and transpiration (Zhao et al., 2020; Fang et al., 2022; Xiao et al., 2023; Pang et al., 2026). However, as the irrigation quota increased, the difference dropped from 23.9% to 5.3%, indicating the potential to compensate for yield reduction through increased irrigation under historical climate conditions (Wang et al., 2023).

Compared with 1981–2020 (+0 °C), the cotton yield in 2021–2060 (+1.6 °C) and 2061–2099 (+2.4 °C) increased by 32.4 and 41.9% with film mulching and by 48.2 and 59.1% without mulching. The results indicates an accelerated growth rate of thermophilic crops like cotton and alterations crop growth cycle as a result of climate warming. It can increase the metabolic and carbon utilization rates of cotton plants, enabling the plant to produce more flowers and buds. These adaptations can help cotton absorb more sunlight and heat for photosynthesis, which contributes to dry matter accumulation (Li et al., 2020a) and eventually increases seed cotton yield in arid oases. Moreover, the rising temperatures during the growth period might enhance more autumn cotton bolls to open and form yield, which can improve the utilization efficiency of ecological and climate resources and the accumulation of dry matter, thereby further increasing yield (Huang and Ji, 2014). The predicted increase in cotton yield in 2061–2099 significantly exceeded that in 2021–2060, possibly attributed to elevated CO_2_ concentration and a pronounced fertilizer effect during 2061–2099, which was consistent with the results of Li et al. (2020a) and Sharma et al. (2024).

Temperature governs key cotton growth processes, including boll development rate, photosynthetic activity, respiration, and evapotranspiration (Bista et al., 2024). Studies indicate that low temperatures (<12°C) during the early growth stage inhibit cotton development, while high temperatures (>35°C) during the mid-growth stage adversely affect pollen viability, boll size, seed number, and the abscission of squares and young bolls, consequently reducing water use efficiency and cotton yield (Li et al., 2020a). Under current climatic conditions in southern Xinjiang, substantial diurnal temperature variations expose cotton to low-temperature stress during approximately one-third of nocturnal hours throughout the growing season. The adoption of drip irrigation under plastic mulch has been shown to elevate soil temperature, preserve soil moisture, and increase the cumulative air temperature required for cotton growth, thereby alleviating the adverse effects of low temperature on cotton productivity (Zumilaiti et al., 2018). Future climate projections suggest a temperature rise of 1.6-2.4°C, which is likely to diminish low-temperature (<12°C) exposure. This modest warming, beneficial during early and late growth stages, can promote biomass accumulation and yield (Sharma et al., 2024). Nevertheless, the yield response of cotton to climate change is expected to vary considerably across regions due to pronounced climatic sensitivity and spatial heterogeneity (Li et al., 2021). These findings call for more granular investigations to clarify sensitivity thresholds and improve predictions of crop responses under future climates (Liu et al., 2025).

Implementing strategies aimed at regulating crop ETc act can positively affect WP, thereby improving sustainable crop production (Busari et al., 2023). Evapotranspiration (ET) is the main form of water consumption in agricultural ecosystems and a pivotal role in water transport within the soil–plant–atmosphere continuum (SPAC), which mainly includes soil evaporation and transpiration from plant stomata (Xu et al., 2023). This study found that soil evaporation of the treatments with film mulching was significantly lower than that without mulching (P<0.05), with an average reduction of 65.2% (1981–2099) compared with the non-mulching treatment. This is because film mulching can block the soil–atmosphere interface, thereby reducing direct evaporation from the soil surface and promoting greater water infiltration compared to evaporation (Yin et al., 2023). The result can effectively reduce the ratio of soil evaporation to evapotranspiration (E/ET) and strengthen the relationship between crop yield and water consumption (Wu et al., 2017). In the case of these mulching methods, the water consumption of cotton increased with an increase in the irrigation quota. A higher irrigation quota can effectively increase soil water storage, establishing the essential foundation for both soil evaporation and plant transpiration, which was consistent with Fan et al. (2013). Furthermore, compared to the 1981–2020 (+0 °C) treatments, our results showed a significant reduction in cotton water consumption under the climate conditions of 2021–2060 (+1.6 °C) and 2061–2099 (+2.4 °C). Specifically, with film mulching, the reductions were 32.0% and 31.7%, respectively, and without mulching, the values were 26.6% and 26.0%, respectively. This results were attributed to the rising temperatures driven by climate change, which lead to a shortened crop growth cycle and a decrease in the cumulative evaporation demand, resulting in a reduction in actual evapotranspiration during the crop growth period (Bouras et al., 2019).

WF quantifies the total freshwater utilization of a product across its entire life cycle, including "blue water", "green water", and "grey water" collected from surface and groundwater resources. Among them, WF_B_ represents the consumption of surface water and groundwater in the production chain, while WF_G_ mainly includes the consumption of soil water in agricultural production (Liu et al., 2023b). This study found that the cotton WF under the historical climate conditions (1981–2020) decreased with an increase in the irrigation quota. This is because the fact that southern Xinjiang relies exclusively on irrigated agriculture, where augmenting irrigation volume substantially enhances cotton yield, thereby reducing the WF. Additionally, the cotton subjected to film mulching exhibited a notably reduced WF compared to the non-mulched cotton, as the absence of surface mulching increased water consumption and reduced cotton yield. The WFs of cotton in 2021–2060 and 2061–2099 were predicted to be significantly lower than those in 1981–2020 because climate change could significantly increase cotton yield and reduce water consumption, thereby reducing the WF (Masasi et al., 2020). This study also found that the WP of cotton in 2021–2060 and 2061–2099 significantly surpassed that in 1981–2020 because climate change could significantly increase cotton yield and hence increase WP.

The future climate data used in this study were derived from the statistically downscaled outputs of a single climate model, BCC-CSM2-MR, within the CMIP6 framework. While this model performs well in simulating climate over China, and its reliability in simulating historical temperatures was verified in this study (**Figure 4a**), it should be noted that the output from any single model carries inherent uncertainties. Future research could incorporate multiple CMIP6 models to quantify the impact of uncertainty in climate change signals on the assessment of cotton production. Furthermore, the detailed simulations conducted for a representative site provide valuable insights for the oasis regions of southern Xinjiang. However, caution is advised when extrapolating these findings to the entire region or to other oasis systems.

## 5 Conclusion

Relative to 1981–2020, the average atmospheric temperatures in 2021–2060 and 2061–2099 could increase by 1.6 °C and 2.4 °C (SSP245), respectively. The rising temperatures could significantly decrease cotton water consumption and WF, boost cotton yield, yield stability and sustainability, and improve WP. Through comprehensive evaluation of TOPSIS based on biomass, yield, soil evaporation, water consumption, WP and WF, we found that under the historical climate conditions of 1981–2020, the irrigation quota of 495 mm (single irrigation 45 mm) for the cotton with film mulching drip irrigation and the irrigation quota of 594 mm (single irrigation 54 mm) for filmless drip irrigation could benefit cotton production. The impact of the mulching method on cotton production under future climate conditions in 2021–2060 and 2061–2099 appeared to be minimal, potentially, allowing for reduced irrigation quotas in cotton production.

## Data Availability

The original contributions presented in the study are included in the article/supplementary material. Further inquiries can be directed to the corresponding authors.
